# Cognitive Impact of Deep Brain Stimulation on Parkinson's Disease Patients

**DOI:** 10.1155/2017/3085140

**Published:** 2017-11-22

**Authors:** Raja Mehanna, Jawad A. Bajwa, Hubert Fernandez, Aparna Ashutosh Wagle Shukla

**Affiliations:** ^1^University of Texas Health Science Center, Houston, TX, USA; ^2^Parkinson's, Movement Disorders and Neurorestoration Program, National Neuroscience Institute, King Fahad Medical City, Riyadh, Saudi Arabia; ^3^Center for Neurological Restoration, Cleveland Clinic, Cleveland, OH, USA; ^4^University of Florida, Gainesville, FL, USA

## Abstract

Subthalamic nucleus (STN) or globus pallidus interna (GPi) deep brain stimulation (DBS) is considered a robust therapeutic tool in the treatment of Parkinson's disease (PD) patients, although it has been reported to potentially cause cognitive decline in some cases. We here provide an in-depth and critical review of the current literature regarding cognition after DBS in PD, summarizing the available data on the impact of STN and GPi DBS as monotherapies and also comparative data across these two therapies on 7 cognitive domains. We provide evidence that, in appropriately screened PD patients, worsening of one or more cognitive functions is rare and subtle after DBS, without negative impact on quality of life, and that there is very little data supporting that STN DBS has a worse cognitive outcome than GPi DBS.

## 1. Introduction

Parkinsonism is defined as bradykinesia with rest tremor or rigidity. Parkinson's disease (PD) is the most frequent cause of parkinsonism and defined by the presence of parkinsonism in the absence of exclusion criteria [1]. With a prevalence of 1 to 2% above the age of 60 years [2], it typically develops between the ages of 55 and 65 years. Pathologically, PD is associated predominantly with the loss of dopaminergic neurons in the substantia nigra. However other brainstem neurons also degenerate in PD, likely contributing to nonmotor impairment [3]. Indeed, PD is a complex syndrome with motor, dermatological, autonomic, neurobehavioral, sensory, and special sense disorders [4]. Many studies have also reported cognitive changes, including impairments in executive functions, language, memory, vision, and psychomotor speed [5–8]. In a cohort comparing 115 patients with newly diagnosed PD to 70 healthy controls, for example, Muslimović et al. [8] reported statistically worse performance in PD patients in most cognitive measures, particularly attention/concentration and executive functions, with 24% of newly diagnosed PD patients (versus 4% of controls) meeting the criteria for cognitive impairment.

Deep brain stimulation (DBS) of the subthalamic nucleus (STN) or the globus pallidus pars interna (GPi) improves quality of life and decreases motor complications in PD and has been approved as such by the Food and Drug Administration in the USA since 2002 [9]. Ablative surgery or DBS of the ventral intermediate (Vim) nucleus of the thalamus is being used for essential and other secondary causes of tremor. However, because it does not address the other cardinal motor symptoms of PD, Vim DBS is rarely used for that disorder [10]. Patients considered for DBS should undergo a thorough multidisciplinary preoperative screening, including a neuropsychological test to rule out dementia or psychiatric comorbidities that could be a contraindication to surgery, in order to avoid implanting poor candidates that will either not benefit enough from DBS or poorly tolerate it [11–15]. However, the cognitive impact of DBS in appropriately selected PD patients is unclear, with various studies producing conflicting results as we will see below. We here endeavor to review the available literature on this subject.

We will first review the available studies on the impact of STN and GPi DBS on each of the following cognitive domains: language, executive function, attention and concentration, memory, visual function, psychomotor and processing speed, and global cognition. We will then review more specifically controlled studies as well as studies directly comparing the cognitive impacts of STN and GPi DBS.

## 2. Methods

Preliminary literature search was conducted through PubMed. Keywords used were “deep brain stimulation”, “parkinson”, and “cognition”. The reference lists of relevant articles were also inspected to locate any potential cited articles that address cognition following STN or GPi DBS. Since Vim DBS is rarely used for PD, and with most of the data on DBS in PD patients stemming from studies on the STN and GPi, studies on Vim DBS in PD patients were not included in our search.

The research terms were intentionally broad to capture as many studies as possible. Studies were reviewed if they were published in the English language and met our minimum inclusion criteria: (1) patients with idiopathic PD who underwent STN or GPi DBS, (2) reporting neuropsychological data after DBS surgery, (3) using at least one standardized neuropsychological instrument, and (4) including at least five subjects followed for a mean of at least 3 months postoperatively.

## 3. Results

### 3.1. Cognitive Changes after DBS

72 studies totaling 2,410 STN DBS patients and 702 GPi DBS patients were reviewed (Tables 1 and 2). Among these, only 20 included statistical correction for multiple analyses or did not require correction because of the statistical method used [16–35], 20 had a control arm formed by PD patients who did not undergo DBS (nonsurgically treated PD patients) [16, 17, 21, 24, 32, 33, 36–49], and 9 compared outcomes between GPi and STN DBS patients [26, 34, 47, 50–55]. All these studies were reviewed with post hoc corrections for multiple analyses when required.

We will first briefly summarize studies that investigated the cognitive outcomes related to STN and GPi and were not designed to directly compare the two targets. There were 62 such studies, totaling 1,913 STN DBS patients and 165 GPi DBS patients.

Our findings are summarized below (Tables 1 and 2).

#### 3.1.1. Language

In the reviewed studies, language was most often assessed using the Boston Naming Test and the subtest Similarities of the Wechsler Adult Intelligence Scale III (WAIS-III), phonemic fluency, and sematic fluency. 

*(1) STN*. Statistically significant worsening in one or more language functions was reported in 27 studies, most often a decrease in fluency, while 3 studies [24, 29, 46] reported improvement in at least one measure of language. There was no significant change in at least one assessed measure of language in 38 studies (Table 1), 21 of which reported no change in any measure of language.

Among the studies reporting worsening, it is unclear if one [56] was corrected for multiple analyses by its authors and, if not, whether such a correction would change the conclusions. Another study [57] was not corrected for multiple analyses, and a post hoc correction was not possible due to the lack of reported *p* value, making it unclear if such a correction would have made the reported worsening statistically not significant.

In all these studies, cognitive outcomes after surgery were compared to baseline preoperative performance (Table 1). In addition, 9 studies compared language performance ON and OFF stimulation [29, 31, 38, 49, 58–62]. After correcting for multiple analyses, a study from Daniele et al. [58] reported worsening of letter verbal fluency compared to the preoperative assessment only at 3 months, when the stimulation was OFF, but not at 6 or 12 months, when the stimulation was ON. This might suggest that a decline in verbal fluency was either more pronounced in the early postoperative stages or attenuated by stimulation. On the other hand, after correction for multiple analyses, Pillon et al. [60] reported no worsening of fluency at 3 months but worsening at 12 months after implant with stimulation ON or OFF. Since patients were assessed ON medications in the study earlier study [58] and, and OFF medication in latter [60], this might suggest a positive synergistic effect of medication and stimulation on fluency. Castner et al. [31] assessed 8 patients ON and OFF stimulation at least 4 months after STN DBS and found that stimulation increased errors in word generation suggesting that STN stimulation might affect the ability to select from many competing lexical alternatives during word generation. In contrast, Silveri et al. [29] studied 12 patients 8 years after STN DBS implant and found an improvement in performance (accuracy and response time) when STN DBS was ON compared to OFF, with less semantic errors, suggesting STN DBS might improve lexical search. The 5 other studies [38, 49, 59, 61, 62] could not elicit any statistical difference between ON and OFF stimulation states.

Most recently, Tröster et al. [35] reported on a total of 136 STN DBS patients followed for 12 months after surgery, divided between 101 receiving constant current stimulation immediately after surgery and 35 starting activation 3 months after surgery. The cognitive assessment at 3 months did indicate some decrease in attention and language even before the device was turned on, with additional deterioration from stimulation. However, the study showed an overall good safety profile of constant current STN DBS.

With regard to the timing of a potential decline in language, Funkiewiez et al. [22] reported worsening of category fluency and total score of fluency at 1 and 3 years compared to baseline, without any further worsening between the two time points.

A parasagittal trajectory for electrode implantation was suggested as a cause of language worsening in some studies [60, 63], as activation of the paracingulate and cingulate sulci was visible on fMRI during word generation [64]. On the other hand, STN DBS might impact the cognitive circuit involved in language as decreased perfusion in the ventral caudate nucleus, anterior cingulate cortex, and left dorsolateral prefrontal cortex (DLPFC) is visible on single photon emission computed tomography (SPECT) in patients with decreased fluency after STN DBS [16]. A more recent study comparing brain positron emission tomographies (PET) in STN DBS patients with and without decreased fluency reported metabolism change in the right middle occipital gyrus, right fusiform gyrus, and right superior temporal gyrus when deficit in phonemic fluency was detected. Decline in semantic fluency however was associated with metabolic changes in the left inferior precentral/postcentral gyrus and the left inferior parietal lobule. Thus, different brain areas were involved in post-DBS deficits in phonemic or semantic fluency in this study, and none of them were frontal areas involved in cognitive functions [65].

On the other hand, Silveri et al. [29] hypothesized that the observed improvement in response time was secondary to improvement of motor components and increased accuracy was due to restoration of the corticostriatal circuits involved in selection processes of a target word among different alternatives. 

*(2) GPi*. Decline in one or more measures of language, most often fluency, was reported in 3 studies totaling 36 patients followed up to 21 months after GPi DBS [25, 50, 66]. While one of these [66] reported this deterioration in both DBS and ablation of GPi, suggesting a consequence of the procedure itself rather than stimulation, this study was not corrected for multiple analyses, and a post hoc correction was not possible due to the lack of reported *p* value, making it unclear if such a correction would have made the reported worsening statistically not significant. In addition, fluency was the only worsened measure of language in 2 of these studies [50, 66].

Three other studies totaling 97 patients followed up to 12 months reported no change in any measure of language (Table 2).

#### 3.1.2. Executive Function

Executive functions were most often assessed using the Wisconsin Card Sorting Test, Trail Making Test Part (Trails B), and Stroop Color-Word Test (Stroop Color-Word).

*(1) STN*. Worsening in at least one measure of executive function was reported in 8 studies. However, one [57] of these was not corrected for multiple analyses, and a post hoc correction was not possible due to the lack of reported *p* value, making it unclear if such a correction would have made the reported worsening statistically not significant. On the other hand, improvement was reported in 3 studies and no statistical difference in any assessed measures of executive function was reported in 36 studies (Table 1).

Executive function ON and OFF stimulations were compared in 10 studies [18, 38, 58–63, 67, 68]. Spatial delayed response was worse with stimulation ON under a high but not low memory load condition in 2 studies [18, 67]. In particular, Alberts et al. [18] reported further worsening in executive functions when multitasking in bilateral compared to unilateral stimulation. On the other hand, one study [63] reported improvement of frontal executive functions with stimulation ON, and the 7 other studies reported no statistically significant change in the assessed measures of executive functions. Additionally, no change in executive function 6 months after surgery with DBS ON, whether ON or OFF medications, was reported in another study [69].

Improvement in executive functions and attention/concentration after STN DBS might be secondary to a decrease in the excessive inhibitory output from the basal ganglia to the frontal cortex [63], and increased activation of the DLPFC on PET scan was reported after STN DBS [70]. 

*(2) GPi*. No statistically significant change in any measure of executive function up to 21 months after GPi DBS was reported in 7 studies, while one study reported improvement of at least one measure of executive function at 6 months [25] (Table 2). One study by Rothlind et al. [47] showed worsening on some measure of executive functions and attention 6 months after GPi DBS, visible at a population level but unlikely to affect individual patients as we will detail in the controlled studies section below.

#### 3.1.3. Attention and Concentration

Attention and working memory were most often assessed using the Stroop Word Test, Trail Making Test part A, the subtests Letter and Number Sequencing and Digit Span of the Wechsler Adult Intelligence Scale III (WAIS-III), the Vienna Test System's simple and choice reaction speed tests, and the Symbol Digit Modalities administration. 

*(1) STN*. All reported measures of attention and concentration (A/C) were improved with stimulation ON compared to OFF in 7 patients [63]. Another series of 12 patients reported similar improvement in some of the reported measures [68]. Comparing 18 patients ON and OFF stimulation at least 4 months after DBS, Castner et al. [30] reported improvement in one measure of attention and no change in another one with ON stimulation. It must be noted that there was no comparison to the pre-DBS level of A/C in these 3 studies to assess if DBS implant, rather than stimulation alone, might be the cause of these changes. Conversely, 8 studies with assessments up to 16 months after STN DBS follow-up reported worsening of at least one measure of A/C, one of which reported no difference between ON and OFF stimulation [62]. Finally, no statistically significant impact of STN DBS implant and/or stimulation on A/C was reported in 21 other series (Table 1), including 2 evaluating patients ON and OFF stimulation [38, 58] and one evaluating patient ON DBS and ON and OFF medications [69].

The missing digit task, used by some studies, specifically activates the posterior premotor cortex and the DLPFC on PET [71], giving a substratum for the observed improvement since the STN projects to these cortical sites [72]. 

*(2) GPi*. Five studies assessing attention and concentration up to 21 months after GPi DBS reported no statistically significant change (Table 2), including no change with DBS ON versus OFF in one study [63]. However, Rothlind et al. [47] reported worsening in some, but not all, measures of A/C 6 months after GPi DBS.

#### 3.1.4. Memory

Memory was most often assessed by the Rey Auditory Verbal Learning Test (RAVLT), the Brief Visuospatial Memory Test, and the Hopkins Verbal Learning Test.

*(1) STN*. Memory improvement 4 months after STN DBS was reported in a series of 8 patients [37]. However, the study was not corrected for multiple analyses, and a post hoc correction was not possible due to the lack of reported *p* value, making it unclear if such a correction would have made the reported improvement statistically not significant. Tang et al. did report such improvement, in a series of 27 patients followed for 12 months [73], in a study corrected for multiple analyses. Conversely, worsening in at least one measure of memory was reported in 5 studies, up to 16 months after DBS (Table 1). However, one of these [57] was not corrected for multiple analyses and a post hoc correction was not possible due to the lack of reported *p* value, making it unclear if such a correction would have made the reported worsening statistically not significant. In addition, there was no difference in memory assessment ON and OFF stimulation in 2 of these studies after correction for multiple analyses [59, 63].

Finally, no statistically significant impact of DBS implant and/or stimulation on memory was reported in 30 other studies (Table 1), including one evaluating patients ON DBS and ON and OFF medications [69]. 

*(2) GPi*. Worsening in one but not all measures of memory was reported in one series of 6 bilateral GPi DBS patients followed for 5 months [74]. However, this study was not corrected for multiple analyses and a post hoc correction was not possible due to the lack of reported *p* value, making it unclear if such a correction would have made the reported worsening statistically not significant. Conversely, no significant change in any measure of memory was detected in 7 other studies totaling 146 patients followed for up to 21 months (Table 2), including 2 studies comparing patients ON and OFF stimulations [60, 63].

#### 3.1.5. Visual Function

Visual function was most often assessed by the subtest Matrix Reasoning of the WAIS-III and Clock Drawing. 

*(1) STN*. Alegret et al. [75] first reported worsening of visuospatial function after STN DBS that was not statistically significant after correction for multiple analyses. However, Smeding et al. [33] reported decrease in visual function in a controlled study of 105 STN DBS patients followed for 12 months. Conversely, 18 other studies, including 2 assessing patients ON and OFF stimulation [38, 62], reported no impact on visual function (Table 1).

*(2) GPi*. Worsening of one but not all measures of visual function was reported in one series of 9 patients followed for 3 months after bilateral GPi DBS [66]. However, this study was not corrected for multiple analyses and a post hoc correction was not possible due to the lack of reported *p* value, making it unclear if such a correction would have made the reported worsening statistically not significant. Conversely, no significant change in any used measure of visual function was detected in 4 studies totaling 44 patients followed up to 21 months (Table 2).

#### 3.1.6. Psychomotor and Processing Speed

The assessment of psychomotor and processing speed is usually included in the assessment of executive and A/C. In some studies though, it was assessed separately, most often assessed using the Stroop Word Test, Trail Making Test part A, the subtest Digit Span of the WAIS-III, and the Symbol Digit Modalities Test oral administration.

*(1) STN*. Improvement in processing and psychomotor speed with STN stimulation ON compared to OFF was reported in 2 studies [63, 68], while another [47] reported worsening of some measures of psychomotor and processing speed when compared to PD patients controls in the medication ON state [47]. Conversely, 13 other studies, including 2 evaluating patients with stimulation ON and OFF [59, 63] and one evaluating patients ON and OFF medications with stimulation ON [69], could not detect significant change after STN DBS (Table 1). 

*(2) GPi*. No significant change in psychomotor and processing speed from GPi implant with or without stimulation could be detected in 5 studies totaling 132 patients [25, 47, 60, 63, 76] (Table 2).

#### 3.1.7. Global Cognition

General cognition was most often assessed by the Mini Mental Status Exam and the Mattis Dementia Rating Scale. 

*(1) STN*. Two series totaling 140 patients evaluating ON stimulation and ON medications reported significant worsening of global cognition 5 years after surgery [23, 76]. However, the reported worsening might have been secondary to the natural evolution of PD [77] since none of these studies had a control arm. On the other hand, a controlled study with 105 STN DBS patients [33] reported worsening of all cognitive domains 12 months after surgery (global cognition, memory, executive function, visual function, attention/concentration, and language).

No significant change was reported in 27 other studies up to 8 years after surgery, including 7 controlled studies comparing a total of 265 STN DBS patients to nonsurgically treated PD patients [16, 17, 32, 40–42, 45, 48] (Table 1). In addition, the incidence of dementia 3 years after bilateral STN DBS in 50 PD patients was estimated at 89 per 1000 by Aybek et al. [19], while Kim et al. [78] had an incidence rate of 35.7 per 1000 person-years in their cohort of 103 STN DBS patients followed for 42 months. Both rates were comparable to the reported incidence in medically managed PD (42.6 to 112 per 1,000) [79]. 

*(2) GPi*. No statistically significant change in global cognition up to 6 months after surgery could be detected in 3 studies totaling 28 patients [25, 66, 74] (Table 2).

### 3.2. Controlled Studies

Because most of the available information is provided by open label uncontrolled series, a major concern is that Parkinson's' disease natural history, rather than DBS, might be the cause of any detected cognitive worsening. It is thus important to consider more attentively the 20 controlled studies available (Table 3).

Among these, seven reported no difference between DBS and non-DBS PD patients. Gironell et al. [36] reported worse semantic verbal fluency in the DBS group when comparing 8 bilateral STN DBS patients 6 months after surgery to 8 age- and stage-matched PD patients who refused surgery. However, this difference was not statistically significant when corrected for multiple analyses, and there was no difference in the other cognitive tasks assessed. A year later, Morrison et al. [38] reported no statistically significant difference at 3 months after surgery between 17 bilateral STN DBS patients and 11 nonsurgically treated PD patients. In addition, within the DBS group, there was no difference between the preoperative assessment and stimulation ON at 3 months, or between stimulation ON and stimulation OFF at 3 months. York et al. [17] reported worse verbal memory in 23 STN DBS patients at 6 months compared to 27 medically managed PD patients. There was no difference in visual memory or other cognitive measures. However, in a follow-up to this study including 19 STN DBS patients and 18 controls 2 years after surgery, Williams et al. [40] reported worsening of some measures of memory, processing, and fluency, but these differences were not significant after correction for multiple analyses. More recently, Sáez-Zea et al. [44] reported no difference 6 months after surgery between 9 bilateral STN DBS patients and 12 nonsurgical PD patients, with worsening of 4 measures of language and attention in each group, out of the 18 cognitive measures assessed. In addition, STN DBS patients had a nonstatistically significant trend to worse phonemic verbal fluency that was significantly correlated with reductions in the L-dopa-equivalent daily dose, suggesting that a decrease in the antiparkinsonian medication might be the actual cause of worse fluency observed after STN DBS. Witt et al. [41] also reported worsening of semantic fluency, but not of letter fluency or other cognitive measures assessed, 6 months after surgery in 31 bilateral STN DBS patients compared to 31 nonsurgical PD patients. However, this difference was not statistically significant after correction for multiple analyses. In a prospective study comparing 11 BL STN DBS and 11 PD controls and 18 healthy controls, Phillips et al. [49] reported improvement in some aspects of language with STN DBS but worsening of others. However, after correction for multiple analyses, these differences were not statistically significant except for a longer reaction time with DBS ON and medication ON compared to DBS OFF and medication OFF, for regular verbs in past tense only, through indirect comparison. However, a direct comparison of these results did not show a statistical significance. Finally, Rukmini Mridula et al. [48] prospectively compared 56 patients who underwent bilateral STN DBS to 53 PD controls in the ON state with a mean follow-up of 23 months, showing no difference in any of the cognitive measures assessed.

In contrast, worsening of some cognitive measures after DBS, sometimes mitigated by improvement of others, was reported in 11 controlled studies. Moretti et al. [46] reported worsening of semantic and syllabic fluency as well as some executive functions, but with an increase in control of linguistic production, 12 months after surgery in 9 bilateral STN DBS patients compared to 9 nonsurgical PD patients. Zangaglia et al. [45] reported worsening of verbal fluency but none of other cognitive measures assessed, 3 years after surgery in 32 STN DBS patients compared to 33 nonsurgical PD patients. In a follow-up publication on that cohort, the authors reported a similar cognitive status 8 years after surgery, concluding that STN DBS was safe from a cognitive standpoint and did not modify the cognitive evolution along the course of the disease [32]. Witt et al. [42] reported worse scores on 2 measures of attention but none of other cognitive measures assessed, 6 months after surgery in 60 bilateral STN DBS patients compared to 63 nonsurgical PD patients, but without comparison to the preoperative baseline. Smeding et al. [43] reported a significantly worse decline in fluency and attention/concentration but none of the other cognitive measures assessed, 6 months after surgery in 99 STN DBS patients compared to 39 nonsurgical PD patients. Cilia et al. [16] reported statistically significant worsening of category fluency but not of phonemic fluency or other cognitive measures assessed, 12 months after surgery in 20 STN DBS patients compared to 12 nonsurgical PD patients. De Gaspari et al. similarly reported decrease in category fluency 12 months after surgery in 12 STN DBS patients compared to 13 nonsurgical PD patients [21]. Last, Castelli et al. [39] reported worsening of phonemic fluency but not of semantic fluency or other cognitive measures assessed, 12 months after surgery in 27 STN DBS patients compared to 31 matched nonsurgical PD patients. In a study comparing 105 STN DBS patients with 40 non-DBS PD controls 12 months after surgery, Smeding et al. [33] reported worsening of all cognitive domains (global cognition, memory, executive function, visual function, attention/concentration, and language) with no worsening in one or more measures of attention/concentration and language. However, disease duration was statistically longer in the STN group, so the possibility of cognitive decline related to the disease rather than DBS cannot completely be ruled out. Regardless, quality of life was significantly better in STN group than in the control group. Whelan et al. [24] compared language 3 months after surgery in 5 bilateral STN PD patients, 16 nonsurgical PD patients, and 16 healthy aged matched subjects. Compared to the nonsurgical PD patients, DBS patients had improvement on the word test-revised but worsening in the accuracy of lexical decisions about words with many meanings and a high degree of relatedness between meanings. The impact of these detailed differential results on the patients' daily life is unclear. More recently, in a prospective unblinded randomized controlled study comparing neuropsychological outcomes between patients treated with bilateral DBS ON stimulation and ON medication (164 patients, 84 implanted in the STN and 80 in the GPi) and patients treated with optimal medication management ON medication (*n* = 116), Rothlind et al. [47] reported significantly greater mean reductions at 6 months in performance on multiple measures of processing speed and working memory in the combined DBS group, as well as higher rates of decline in neuropsychological test performance in this group [47]. Decline by multiple indicators in two or more cognitive domains was seen in 11% of the DBS patients and 3% of the medically managed patients. This multidomain cognitive decline was associated with less beneficial change in subjective ratings of everyday functioning and quality of life. However, the authors noted that the majority of individual patients receiving DBS did not display changes on individual measures or combinations of measures that would clearly distinguish them from patients treated with optimal medication management and in fact showed, for most of them, a balance of isolated declines and improvements in test performance similar to the pattern observed in the optimal medication management arm. In other words, worsening of some neuropsychological tests after DBS was observed at a population level but was unlikely to affect individual patients in the majority of the cases.

However, Hilker et al. [37] reported significant improvement in verbal and nonverbal long-term memory 4 months after surgery in 8 bilateral STN DBS PD patients compared to 10 healthy matched controls suggesting STN DBS might in fact improve memory circuits. The study was not corrected for multiple analyses and a post hoc correction was not possible due to the lack of reported *p* value, making it unclear if such a correction would have made the reported improvement statistically not significant.

In summary, 10 of the 20 available controlled studies reported statistically significant worsening on some cognitive measures after bilateral STN DBS and 2 reported improvement in some and worsening in other cognitive measures. Different subtypes of fluency (semantic, phonemic, and category) worsened in some studies but not others. Worsening of attention was also reported in more than one controlled study. On the other hand, one controlled study reported improvement and 7 did not detect any cognitive difference between STN DBS and non-DBS PD patients.

### 3.3. Target Selection

Currently, most DBS centers prefer to implant in the GPi in PD patients with mild cognitive impairment, out of fear that STN DBS would cause more cognitive side effects. There is indeed more data in the literature reporting cognitive worsening after STN DBS than GPi DBS, but this data is markedly imbalanced as the studies detailed above have evaluated 1,777 STN DBS patients but only 165 GPi patients. It is important therefore to look more attentively at head-to-head comparison between the 2 targets.

Head-to-head comparison of the cognitive impact of STN and GPi DBS was reported in 9 studies to our knowledge, with a total of 581 STN and 617 GPi patients [26, 34, 47, 50–55] (Table 4). Only one of these [51] reported correction for multiple analyses. After corrections were applied when needed, the following studies revealed a difference between the 2 groups.

Weaver et al. [53] followed 159 patients for 3 years after surgery and reported worsening of one out of 4 memory measures after STN DBS compared to GPi DBS. The authors suggested that this difference might be secondary to a larger decrease in dopamine replacement doses in the STN group. Although Rothlind et al. [47] reported slightly greater reductions in some aspects of processing speed in the STN group and greater reductions in verbal learning and recall in participants in the GPi group, the 2 groups were deemed similar overall. Odekerken et al. [54] reported a bigger negative change in the STN group 12 months after surgery, in 4 out of 11 measures of attention, out of the 24 cognitive measures assessed. However, the frequency of cognitive decline and the quality of life were similar between the 2 groups. Of note, the authors also reported that an older age at surgery was associated with a higher risk of cognitive decline (62.4 versus 58.4 years). On the other hand, in a 36-month follow-up to this study, Odekerken et al. [34] reported no difference between the 2 groups on a composite score for cognition, mood, and behavior but reported better OFF drug motor symptoms and functioning in the STN group, as well as bigger medication reduction in that group and a higher rate of repeat surgery in the GPi group.

In summary, and after correction for multiple analyses, only 2 out of 9 studies [53, 54], totaling 126 STN and 145 GPi patients, reported worse outcome in the STN group in some measures of attention or memory. However, quality of life was similar in the 2 groups. Interestingly, these studies did not report any worse decline in language, fluency, or executive function in the STN group, as would have been expected from the open label and controlled studies. Overall, these data do not support favoring GPi over STN for fear of cognitive complications from the latter [80] in properly screened PD patients.

## 4. Discussion

Studies on cognitive changes after DBS in PD patients have reported different and sometimes opposite results. However, any change revealed by cognitive tests is likely subtle as detected cognitive worsening on specialized tests was usually not reported by patients, caregivers, or healthcare providers [25, 81]. In addition, quality of life measures in these patients showed improvement, even when cognitive worsening was detected [33, 53, 54, 58, 82].

Our findings confirm results from a recently published meta-analysis by Combs et al. [81] including 38 articles with an aggregated sample size of 1622 patients. The authors searched keywords and had selection criteria similar to ours, with the exception of needing sufficient report of study results to allow for an effect size to be calculated. These additional criteria might explain the lower number of studies included in the meta-analysis compared to our current review. Among the articles reviewed, 30 included STN DBS patients only, 5 reported on GPi DBS only, and 3 compared GPi and STN DBS. Combs et al. reported a small decline in psychomotor speed, learning and memory, fluency, attention/concentration, executive functions, and general cognition after STN DBS. GPi DBS patients had small changes in attention/concentration and fluency. The authors warned against concluding that GPi DBS would be cognitively safer than STN DBS, because of the small number of GPi DBS studies included.

Kumar et al. [83] suggested that variability in lead placement inside the target might explain the variation in the results of different studies. Tsai et al. [84] suggested that an active contact anteriorly located within the ventral STN could cause the neuropsychological effects reported in chronic STN DBS. York et al. [85] suggested that, in addition to the precise location of the active electrode inside the STN, a surgical trajectory through the frontal lobe might also influence the cognitive outcome. Indeed, Witt et al. [41] reported a higher risk of decline in working memory performance and global cognition associated with a trajectory intersecting the caudate nucleus. On the other hand, Smith et al. [86] could not find any correlation between decline in verbal fluency and any of age at surgery, number of intraoperative microelectrode penetrations, coordinates of the lead tip, or active stimulation site in a series of 28 STN DBS patients. Larger series have yet to duplicate these results. Trépanier et al. [87] also suspected variations in the characteristics of the patients selected for surgery between different centers (age, preoperative cognitive status, and comorbidity with other conditions such as psychiatric disorders) to explain conflicting conclusions from different studies.

In addition, outcome can also be influenced by stimulation parameters. Wojtecki et al. [88] reported a frequency-dependent modulation of cognitive circuits involving the STN, with low frequency (10 Hz) STN DBS improving verbal fluency compared to no stimulation and high frequency (130 Hz) STN DBS causing a nonsignificant trend towards worsening of fluency compared to no stimulation. Schoenberg et al. [89] reported improvement in cognitive test scores with increased amplitude and pulse width of the stimulation in 20 bilateral STN PD patients.

The respective contribution of lead implant and stimulation to post-DBS cognitive change is difficult to ascertain. The COMPARE trial [51] reported worsening of letter verbal fluency that persisted even when DBS was turned OFF, suggestive of a surgical rather than a stimulation-induced effect. On the other hand, Tröster et al. [35] reported worsening of measures of language and attention even before DBS was tuned ON, with further worsening after activation.

Studies assessing cognitive change after DBS for PD can have the following limitations. First, most the available studies lack a control arm of non-DBS treated PD patients, and a reported cognitive decline might thus be caused by the natural evolution of PD rather than DBS. Second, a reported cognitive improvement may stem from practice effect in the case of repeated cognitive assessment [58]. Using parallel forms of cognitive tasks might mitigate this practice effect, but it may be logistically difficult. Alternatively, cognitive assessments could be repeated at relatively long intervals [58]. Third, all studies did not assess patients in the same pharmacological condition, with most studies assessing patients ON antiparkinsonian medications, some studies assessing them OFF antiparkinsonian medications [38, 51–53, 63, 67, 75], and some other studies assessing them in a nonhomogenous way [25]. Some authors did not specify the medication and/or stimulation status of the patients at the time of cognitive evaluation [27, 28, 35]. Finally, cognitive worsening after DBS might be at least partially secondary to a postoperative reduction in antiparkinsonian medications, which is seen more after STN DBS than GPi DBS [9, 34]. A uniform assessment ON stimulation and OFF medications could minimize this confounding factor. However, severity of symptoms OFF medications might render such a preoperative assessment impossible in some patients.

## 5. Conclusion

After reviewing the available studies assessing cognitive changes after STN and GPi DBS in PD patients, we arrive at the following suggestions. (1) In PD patients who are adequately screened for surgery, worsening of one or more cognitive functions is rare after DBS, with available studies reporting conflicting results. (2) Any change revealed by cognitive tests is likely subtle as a detected cognitive worsening on specialized tests is usually not reported by patients, caregivers, or healthcare providers. Furthermore, there is an improvement in quality of life after DBS, even when cognitive worsening is detected. (3) Worse cognitive outcome after STN DBS compared to GPi DBS was reported only in 2 out of 9 randomized trials. As such, fear of cognitive worsening should not systematically exclude STN as a potential DBS target. (4) Ideally, future studies on this topic should include controls for the natural evolution of PD. This can be done by using nonsurgically treated PD patients matched for all clinical and demographic variables. In addition, DBS patients should be assessed ON and OFF stimulation, thus providing direct comparison of the stimulatory effects while controlling for the effects of surgery. (5) Additional reports on anatomoclinical correlation of cognitive worsening after DBS would help improve surgical planning to avoid sensitive structures.

## Figures and Tables

**Table 1:** Studies assessing cognitive change in PD patients after STN DBS.

Author, year	*N*	F/u (mo)	Controlled	Status of stimulation/medication at cognitive assessment	Improved cognitive measure(s)	Worsened cognitive measure(s)	Unchanged cognitive measure(s)
Alberts et al., 2008 [18]	8	N/A	No	UL, BL, ON, OFF/ON	None	E	None

Alegret et al., 2001 [75]	15	3	No	ON/OFF	None	None	E, PS, L, M, V

Ardouin et al., 1999 [25]	49	3–6	No	ON/inconstant	E	L	GC, E, PS

Asahi et al., 2014 [27]	11	12	No	Unspecified	None	None	GC, A/C, M, L, V

Castelli et al., 2006 [56]	72	15	No	ON/-	E	L	E, L, M

Castelli et al., 2007 [90]	19	17	No	ON/ON	None	L	E, V, M, L,

Castelli et al., 2010 [39]	27	12	Yes	ON/ON	None	L	E, A/C, M, L

Castner et al., 2007 [30]	18	At least 4	No	ON and OFF/ON	A/C	None	A/C

Castner et al., 2008 [31]	8	At least 4	No	ON and OFF/ON	None	L	L

Cilia et al., 2007 [16]	20	12	Yes	ON/ON	None	L	GC, L, E, A/C

Contarino et al., 2007 [91]	11	60	No	ON/ON	None	None	L, V, M, E

Daniele et al., 2003 [58]	20	12	No	ON or OFF/ON	None	L	GC, L, E, A/C, M

De Gaspari et al., 2006 [21]	12	12	Yes	ON/ON	None	L	L, GC, E

Dujardin et al., 2001 [92]	9	3	No	ON/ON	None	None	GC, E, M, PS, L

Erola et al., 2006 [93]	19	12	No	ON/ON	None	L	GC, E, PS

Fasano et al., 2010 [57]	16	96	No	ON/ON	None	E, L, M	GC, M, E, L

Fraraccio et al., 2008 [62]	15	16	No	ON and OFF/ON	None	A/C	E, A/C, M, L, V, CG

Funkiewiez et al., 2003 [94]	50	12^a^	No	ON/OFF	None	None	GC, E

Funkiewiez et al., 2004 [22]	70	36	No	ON/69% OFF	None	L	GC, E, M, PS

Gironell et al., 2003 [36]	8	6	Yes	ON/ON	None	None	L, E, A/C, M, V, PS

Hälbig et al., 2004 [59]	12	16	No	ON and OFF/ON	None	None	PS, M, GC, E, L

Heo et al., 2008 [95]	46	12	No	ON/ON	None	None	GC, A/C, M, L, E

Hershey et al., 2004 [67]	24	7^b^	No	ON and OFF/OFF	None	E	None

Hilker et al., 2004 [37]	8	4	Yes	ON/-	M	None	GC, E, L, A/C, M, V

Jahanshahi et al., 2000 [63]	7	12	No	ON and OFF/OFF	E, A/C, PS	M	None

Kim et al., 2014 [78]	103	42^b^	No	ON/ON	None	GC, but similar incidence to incidence of PDD	None

Krack et al., 2003 [20]	42	60	No	ON/ON	None	None	GC, E

Krugel et al., 2014 [96]	14	N/A	No	ON/ON	None	None	L

Lhommée et al., 2012 [97]	63	3	No	ON/ON	None	L	GC, E

Limousin et al., 1998 [70]	24	12	No	ON/OFF	None	None	E, L, V, PS

Moretti et al., 2003 [46]	9	12	Yes	ON/ON	L	L, E	E, L, A/C, M, V

Moro et al., 1999 [98]	7	9	No	ON/ON	None	None	GC, E, L, M

Morrison et al., 2004 [38]	17	3	Yes	ON and OFF/OFF	None	None	L, A/C, M, E, V

Rukmini Mridula et al., 2015 [48]	50	23^b^	Yes	ON/ON	None	None	GC, A/C

Page and Jahanshahi, 2007 [68]	12	N/A	No	ON and OFF/ON	PS, A/C	None	PS, A/C, E

Perozzo et al., 2001 [69]	20	6	No	ON/ON and OFF	None	None	E, A/C, M, PS

Phillips et al., 2012 [49]	11	13.8^b^	Yes	ON and OFF/ON and OFF	None	None	L

Pillon et al., 2000 [60]	63	12	No	ON and OFF/75% OFF	None	L	E, PS, L, M

Rothlind et al., 2007 [50]	15	21	No	ON/ON	None	L	A/C, E, L, V, M

Rothlind et al., 2015 [47]	84	6	Yes	ON/ON	None	E, A/C, PS (see text)	E, M, A/C, L, PS

Sáez-Zea et al., 2012 [44]	9	6	Yes	ON/ON	None	L, A/C	A, M, V, E, L

Saint-Cyr et al., 2000 [99]	11	12	No	ON/ON	None	L	E, L, M, A/C, V

Saint-Cyr and Albanese, 2006 [82]	99	6	No	ON/ON	None	L, E	E, L, A/C, M, PS

Schüpbach et al., 2005 [23]	37	60	No	ON/ON	None	CG, E	None

Silveri et al., 2012 [29]	12	96	No	ON and OFF/ON	L	None	None

Smeding et al., 2011 [33]	105	12	Yes	ON/ON		GC, E, L, V, M, A/C	L, A/C

Smeding et al., 2006 [43]	99	6	Yes	ON/ON	None	L, A/C	L, M, V, A/C

Tang et al., 2015 [73]	27	12	No	ON/ON	M	L	GC, M, V, A/C, E, L

Tremblay et al., 2015 [28]	8	At least 7 wks	No	OFF DBS and then ON DBS/unspecified med status	None	L	L

Trepanier et al., 2000 [87]	9	6	No	ON/ON	None	None	A/C, M, V, L, E

Whelan et al., 2003 [24]	5	3	No	ON/ON	L	L	None

Williams et al., 2011 [40]	19	24	Yes	ON/ON	None	None	GC, M, E, A/C, L, V, PS

Witt et al., 2004 [61]	23	12	No	ON and OFF/ON	None	None	L, E, GC

Witt et al., 2008 [42]	60	6	Yes	ON/ON	None	A/C	GC, E, L, A/C

Witt et al., 2013 [41]	31	6	Yes	ON/ON	None	None	GC, A/C, L

Yágüez et al., 2014 [100]	30	9	No	ON/ON	None	L, M	GC, M, L, V, E

York et al., 2008 [17]	23	6	Yes	ON/ON	None	M	GC, E, A/C, M, L, V, PS

Zangaglia et al., 2009 [45]	32	36	Yes	ON/ON	None	L	GC, M, E, A/C

Zangaglia et al., 2012 [32]	30	96	Yes	ON/ON	None	L	GC, M, E, A/C

PD: Parkinson's disease; STN: subthalamic nucleus; *N*: number of patients; mo: months; A/C: attention/concentration; E: executive; GC: global cognition; L: language; M: memory; PS: psychomotor/processing speed; V: visual. ^a^Median; ^b^mean. *Note*. Multiple tests were performed for each domain in each study, and often a few of these showed a difference while other tests assessing the same domain did not. This explains why the same domain might appear more than once and under different results for the same study. Adapted from Mehanna [101] with permission from the author.

**Table 2:** Studies assessing cognitive change in PD patients after GPi DBS.

Author, year	*N*	F/u (mo)	Controlled	Status of stimulation/medication at cognitive assessment	Improved cognitive measure(s)	Worsened cognitive measure(s)	Unchanged cognitive measure(s)
Ardouin et al., 1999 [25]	13	3–6	No	ON/inconsistent	E	L	GC, E, PS
Jahanshahi et al., 2000 [63]	6	12	No	ON and OFF/OFF	None	None	E, A/C, PS, M,
Pillon et al., 2000 [60]	13	12	No	ON and OFF/75% OFF	None	None	E, PS, L, M
Trépanier et al., 2000 [87]	4	6	No	ON/ON	None	None	A/C, M, V, L, E
Rothlind et al., 2007 [50]	14	21	No	ON/ON	None	L	A/C, E, L, V, M
Fields et al., 1999 [74]	6	5	No	ON/ON	M	None	GC, E, A/C, V, L, M
Tröster et al., 1997 [66]	9	3	No	ON/ON	None	V, L	GC, E, A/C, V, M, L
Tröster et al., 2017 [35]	136	12	No	ON/not specified	None	L, A/C	GC, A/C, E, M
Vingerhoets et al., 1999 [76]	20	3	No	ON/ON	None	None	M, V, E, PS
Rothlind et al., 2015 [47]	80	6	Yes	ON/ON	None	E, A/C	E, M, A/C, L, PS

PD: Parkinson's disease; GPi: globus pallidus interna; *N*: number of patients; mo: months; A/C: attention/concentration; E: executive; GC: global cognition; L: language; M: memory; PS: psychomotor/processing speed; V: visual. *Note*. Multiple tests were performed for each domain in each study, and often a few of these showed a difference while other tests assessing the same domain did not. This explains why the same domain might appear more than once and under different results for the same study. Adapted from Mehanna [101] with permission from the author.

**Table 3:** Controlled studies assessing cognitive change in PD patients after DBS.

Author, year	*N* of DBS patients	F/u (mo)	Lead location	Status of stimulation/medication at cognitive assessment	Cognitive performance(s) improved in DBS group	Cognitive performance(s) worsened in DBS group	Cognitive performance(s) not different between DBS and control group
Castelli et al., 2010 [39]	27	12	STN	ON/ON	None	L	E, A/C, M, L

Cilia et al., 2007 [16]	20	12	STN	ON/ON	None	L	GC, L, E, A/C

De Gaspari et al., 2006 [21]	12	12	STN	ON/ON	None	L	GC, E, L

Gironell et al., 2003 [36]	8	6	STN	ON/ON	None	None	L, E, A/C, M, V, PS

Hilker et al., 2004 [37]	8	4	STN	ON/-	M	None	GC, E, L, A/C, M, V

Moretti et al., 2003 [46]	9	12	STN	ON/ON	L	L, E	E, L, A/C, M, V

Morrison et al., 2004 [38]	17	3	STN	ON and OFF/OFF	None	None	L, A/C, M, E, V

Rukmini Mridula et al., 2015 [48]	50	23^a^	STN	ON/ON	None	None	GC, A/C

Phillips et al., 2012 [49]	11	13.8^a^	STN	ON and OFF/ON and OFF	None	None	L

Rothlind et al., 2015 [47]	281	6	STN *n* = 84 and GPI *n* = 80	ON/ON	None	A/C, E, PS, see text	A/C, E, L, M, PS

Sáez-Zea et al., 2012 [44]	9	6	STN	ON/ON	None	None	A, M, V, E, L, A/C

Smeding et al., 2011 [33]	105	12	STN	ON/ON	None	GC, E, L, V, M, A/C	L, A/C

Smeding et al., 2006 [43]	99	6	STN	ON/ON	None	L, A/C	L, M, V, A/C

Whelan et al., 2003 [24]	5	3	STN	ON/ON	L	L	None

Williams et al., 2011 [40]	19	24	STN	ON/ON	None	None	GC, M, E, A/C, L, V, PS

Witt et al., 2008 [42]	60	6	STN	ON/ON	None	A/C	GC, E, L, A/C

Witt et al., 2013 [41]	31	6	STN	ON/ON	None	None	GC, A/C, L

York et al., 2008 [17]	23	6	STN	ON/ON	None	M	GC, E, A/C, M, L, V, PS

Zangaglia et al., 2009 [45]	32	36	STN	ON/ON	None	L	GC, M, E, A/C

Zangaglia et al., 2012 [32]	30	96	STN	ON/ON	None	L	GC, M, E, A/C

PD: Parkinson's disease; STN: subthalamic nucleus; *N*: number; mo: months; A/C: attention/concentration; E: executive; GC: global cognition; L: language; M: memory; PS: psychomotor/processing speed; V: visual. ^a^mean. *Note*. Multiple tests were performed for each domain in each study, and often a few of these showed a difference while other tests assessing the same domain did not. This explains why the same domain might appear more than once and under different results for the same study. Adapted from Mehanna [11] with permission from the author.

**Table 4:** Studies comparing cognitive outcomes between GPi and STN DBS in PD patients.

Author, year	*N* STN/GPi	Laterality	F/u (mo)	Status of stimulation/medication at cognitive assessment	Cognitive measures assessed	Differences between GPI and STN
Boel et al., 2016 [55]	63/65	BL	36	ON/ON	GC, A/C, E, M, L	None

Follett et al., 2010 [52]	147/152	BL	24	ON/OFF	GC, L, V, E, M	None

Odekerken et al., 2013 [26]	63/65	BL	12	ON/integrated ON and OFF	Composite test	None

Odekerken et al., 2015 [54]	56/58	BL	12	ON/ON	A/C, E, M, L, V	4/11 measures of A/C worse with STN

Odekerken et al., 2016 [34]	43/47	BL	36	ON/ON	A/C, E, M, composite score	None

Okun et al., 2009 [51]	22/23	UL	7	ON/OFF	L	None

Rothlind et al., 2007 [50]	19/23	UL	6	ON/ON	A/C, E, L, V, M	None

Rothlind et al., 2007 [50]	14/15	BL	21	ON/ON	A/C, E, L, V, M	None

Rothlind et al., 2015 [47]	84/80	BL	6	ON/ON	A/C, E, GC, L, M	None overall, E worse with STN, M worse with Gpi

Weaver et al., 2012 [53]	70/89	BL	36	ON/OFF	GC, L, V, E, M	M worse with STN

PD: Parkinson's disease; GPi: globus pallidus interna; STN: subthalamic nucleus; *N*: number of patients; mo: months; UL: unilateral; BL: bilateral; A/C: attention/concentration; E: executive; GC: global cognition; L: language; M: memory; V: visual. Adapted from Mehanna [101] with permission from the author.

## Abbreviations

DBS: Deep brain stimulation
STN: Subthalamic nucleus
GPi: Globus pallidus interna
PD: Parkinson's disease
LID: Levodopa induced dyskinesias
DLPFC: Dorsolateral prefrontal cortex.
